# Psychological distress in cancer patients assessed with an expert rating scale

**DOI:** 10.1038/sj.bjc.6604420

**Published:** 2008-06-24

**Authors:** P Herschbach, K Book, T Brandl, M Keller, G Lindena, K Neuwöhner, B Marten-Mittag

**Affiliations:** 1 Division of Psychosocial Oncology, Department and Outpatient Clinic of Psychosomatic Medicine and Psychotherapy, Technical University of Munich, Munich, Germany; 2 Center for Psychosomatic Medicine, Bad Wiessee, Germany; 3 Division of Psychooncology, Department of Psychosomatic and General Internal Medicine, University of Heidelberg Medical Center, Germany; 4 CLARA Clinical Analysis, Research and Application, Kleinmachnow, Germany; 5 Clinic Dr Hancken, Center for Palliative Medicine, Stade, Germany

**Keywords:** psychosocial distress, expert rating scale, psycho-oncology

## Abstract

The purpose of this study was to investigate psychosocial stress in a large sample of cancer patients using an expert rating scale. Specific aims were to analyse the relevance of setting variables (type of clinic, contact initiative, therapy) and gender. A total of 6365 patients were assessed in 105 institutions. Univariate and multivariate statistical analysis of setting variables indicated that patients treated in palliative care settings showed highest distress scores compared to patients recruited from hospitals and outpatient clinics (*P*<0.001). Significant differences were also found for contact initiative (*P*<0.001); lowest distress was found in patients who were recruited by routine contact. Patients who asked for psychosocial support or who were recruited by the medical staff showed the highest distress scores. The analysis of therapy groups showed that patients receiving radiotherapy or surgery were not more distressed than patients without therapy. The most distressing treatment was chemotherapy. Gender had differential effects on clinic type (*P*<0.001) and contact initiative (*P*<0.001) but not on treatment and diagnosis. Expert rating scales are an important complement for self-assessment questionnaires to evaluate psychological distress of cancer patients in psychosocial studies as well as in routine medical care.

The burden of cancer patients is well documented. Around 30% of all patients show psychosocial distress (Ibbotson *et al*, 1994; Ford *et al*, 1995; Van't Spijker *et al*, 1997; Payne *et al*, 1999; Härter *et al*, 2001; Zabora *et al*, 2001; Herschbach *et al*, 2004a; Bultz and Carlson, 2006; Sellick and Edwardson, 2007). However, prevalence rates depend on the way we define and assess distress. We may examine the prevalence of mental disorders with standardised interviews (Härter *et al*, 2001) or with questionnaires such as the Brief Symptom Inventory (Derogatis, 1993) or the Hospital Anxiety and Depression Scale (Zigmond and Snaith, 1983; Sellick and Edwardson, 2007). We also use global health scales (General Health Questionnaire; Goldberg and Williams, 1988), cancer-specific distress scales (Roth *et al*, 1998; Herschbach *et al*, 2004a), and quality-of-life inventories that are cancer specific (EORTC Quality of Life Questionnaire; Aaronson and the EORTC QoL-study-group, 1991; Functional Assessment of Cancer Therapy; Cella *et al*, 1993) or generic (Spilker, 1990).

Till today, no expert rating scale focused specifically on psychosocial distress in cancer patients. The only existing rating scales are the Karnofsky-Index (Karnofsky *et al*, 1948) and Spitzer-Index (Spitzer *et al*, 1981), which assess physical functioning. The first expert rating scale that assesses psychological distress of cancer patients has recently been developed, the Basic Documentation for Psycho-Oncology (PO-Bado) (Herschbach *et al*, 2004b; Knight *et al*, 2008). This is an instrument applicable for all types and stages of cancer. The PO-Bado does not provide psychiatric diagnoses; however, it enables health-care professionals in all settings to carry out a disease-specific and standardised assessment of distress as a basis for decisions and appropriate interventions (Knight *et al*, 2008).

The use of expert rating scales has some advantages compared to self-assessment questionnaires. First, they allow one to take the overall impression of the patient into account, including non-verbal behaviour, and to examine aspects of illness experience that are not assessed by self-assessment questionnaires, such as denial. It is defined as an ‘… adaptive strategy to protect against overwhelming events and feelings’ (Vos and de Haes, 2007) and a very important and frequent coping strategy in the illness experience. Second, expert rating scales can be used for patients who are unable to answer questionnaires due to mental or physical problems.

The purpose of this paper is to analyse distress of cancer patients on the basis of an expert rating. We were particularly interested in the relevance of setting variables (type of hospital, contact initiative, and type of treatment) and gender for the psychosocial stress profile. Furthermore, the influence of diagnosis on psychosocial stress was examined.

## Materials and methods

### Participants

The total sample included 6365 cancer patients who were treated and assessed between 2003 and 2006. Among them, 4743 patients were recruited from 25 hospitals, 6 rehabilitation clinics, and 4 outpatient clinics. An additional 1613 patients were recruited from an ongoing study on palliative care including 42 palliative care wards, 6 cancer wards, 7 hospices, and 15 outpatient clinics (HOPE-study; Lindena *et al*, 2005).

### Procedure

Patients were recruited in three different ways: they asked for psychosocial support themselves, they were contacted by doctors and nurses, or they were recruited through routine contact. The PO-Bado interviews and ratings were conducted once at the beginning of medical treatment by clinical psychologists and doctors (*n*=58) who received a manual and an interview guideline. The manual gives detailed definitions of each item and criteria for the item ratings. The interview guideline includes instructions for a structured assessment and sample questions for each item.

### Measures

The PO-Bado assesses the subjective cancer-related experience of the patient within the last 3 days. The interview takes about 20–30 min. It consists of the two dimensions physical and psychological distress. The physical dimension contains four items: limitations of everyday activities, fatigue, pain, and one item for other physical complaints. The psychological dimension has eight items: anxiety/worries, depression/grief, helplessness, shame/loss of self-esteem, mood swings, sleep disturbance, cognitive impairment, and other psychological distress. Each item is rated on a five-point Likert scale from 0 (not at all distressing) to 5 (very much distressing), indicating how much the patient suffers subjectively from these illness aspects. Three scores can be evaluated, a physical score (score for 0–16), a psychological distress score (0–32), and a total score (0–48). Additionally, patient's socio-demographic and medical characteristics as well as current treatments are documented.

The psychometric evaluation (Knight *et al*, 2008) showed satisfactory results for internal consistency with Cronbach's *α* coefficient of 0.70 for the physical distress items and *α*=0.85 for the psychological distress items. Inter-rater reliability (intraclass correlation coefficient) varied between 0.79 and 0.85 for the somatic items and between 0.75 and 0.90 for the psychological items. For convergent validity, Spearman's rank correlation coefficients were calculated for the QSC (Questionnaire on Stress in Cancer Patients QSC R 23 – revised version; Herschbach *et al*, 2004a) and the HADS (Hospital Anxiety and Depression Scale; Zigmond and Snaith, 1983) and showed substantial correlations with the PO-Bado. The analyses of the discriminant validity demonstrated the ability of the PO-Bado to differentiate patient groups according to gender, disease status, type of treatment, and functional status.

### Data analysis

Group differences of PO-Bado distress scores (total scores) were investigated with *t*-tests and F-tests. To investigate the effects of gender, clinical setting variables (type of clinic, contact initiative, therapy), and diagnoses on distress scores, four two-factor analyses of variance were conducted for type of clinic, contact initiative, therapy, and diagnoses as the first factors and with gender as the second factor. Type III sums of squares were used.

The results of ANOVA for each independent variable are reported first, followed by the mean distress scores for males and females. To account for multiple comparisons, *P*-values were multiplied by the number of tests when appropriate.

According to the large sample size, statistical significance was assumed at *P*<0.01. All statistical analyses were carried out with the Statistical Package for Social Sciences (Version 14.0).

## Results

First, the characteristics of the sample will be described, followed by the presentation of single stress items for males and females. Second, distress patterns for males and females in different settings will be investigated, followed by the analysis of distress in males and females in the diagnostic subgroups.

### Sample

The total sample included 6365 cancer patients. Table 1 shows the demographic characteristics of the sample for males and females. Of the total sample, 66% were females, with a mean age of 59.5 years (s.d. 14.0). Seventy-one per cent of the patients had a partner. The largest proportion of patients was on sick leave (30%) or retired (45%).

Medical data are presented in Table 2. The most frequent diagnoses of the patients were breast cancer (34%), haematological neoplasias (12%), and tumours of the gastrointestinal tract (18%); 6% of the sample had cancer of unknown cause, rare, or other cancer diagnoses. The most frequent diagnoses for men were tumours of the gastrointestinal tract (26%) and haematological neoplasias (19%). For women, breast cancer (52%), gynaecological carcinomas (12%), and tumours of the gastrointestinal tract (12%) were the most frequent.

Nearly half of the total sample (43%) had metastases and 21% had relapses in their clinical course. The mean duration of cancer (time from diagnosis to inclusion into the study) was 22 months; 11% of patients had a disease duration of more than 5 years.

In terms of treatment within the last 2 months, 40% of the patients had surgical treatments, 10% in combination with either chemotherapy or radiotherapy or both therapies. Twenty-three per cent received chemotherapy only and 13% of the sample received chemotherapy in combination with surgery and/or radiotherapy. Women received more surgeries and fewer chemotherapies than men. Nineteen per cent of the sample had no treatment during the last 2 months. In terms of ‘contact initiative’, 24% of the patients asked for psychosocial support, 27% were contacted by doctors or nurses, and 49% were recruited through routine contact. The majority of patients were recruited from university and general hospitals (68%), followed by patients from palliative care (26%).

### Psychological distress

**Figure 1:**
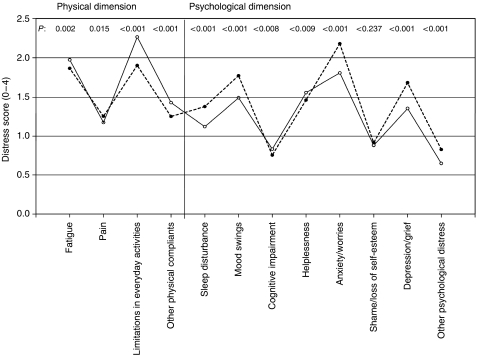
Distress scores of PO-Bado single items for male (solid line) and female patients.

Figure 1 shows the mean distress score for the 12 single items for males and females.

The distress pattern indicates a tendency for higher distress for males in the physical dimension and for females in the psychological dimension. The highest distress scores were ‘limitations in everyday activities’ for males and ‘anxiety/worries’ for females. All gender differences were significant with the exception of ‘shame/loss of self-esteem’. The total distress score for the sample is 17.03 (s.d. 9.53) with a significant difference between males (16.55, s.d. 9.55) and females (17.25, s.d. 9.50; *P*=0.005).

An ANOVA was conducted for type of clinic and gender as the two independent factors and distress as the outcome variable. The analysis revealed a significant main effects for type of clinic (F=124.45, *P*<0.001) and gender (women>men; F=18.36, *P*<0.001) with a significant interaction (F=19.56, *P*<0.001). Thus, distress for male and female patients differs according to the type of clinic.

Table 3 shows the mean distress scores for male and female patients within the different clinic types. Highest scores for the total sample were found in patients treated in palliative care units and hospices. The lowest scores were found in patients treated in general hospitals. Comparisons of mean distress in various types of clinics were conducted separately for men and women and showed significant differences within males as well as within women (*P*<0.001). Comparisons between males and females within each type of clinic showed significantly higher scores in women treated in university clinics and rehabilitation clinics.

The second ANOVA was conducted for contact initiative and gender. It showed a significant main effect for contact initiative (F=329.45, *P*<0.001) and gender (women>men; F=17.77, *P*<0.001) with a significant interaction (F=7.05, *P*=0.001). Distress scores in the three categories of contact initiative differed significantly within males (*P*<0.001) and females (*P*<0.001) (Table 4). Patients recruited by routine contact showed the lowest distress scores; the highest distress scores were found for patients who asked for psychosocial support. A comparison of distress means between males and females within each category showed that women report significantly higher distress in the category patient initiative and routine contact (*P*<0.001). No significant gender differences were found in patients who were recruited by doctors and nurses.

In the investigation of the effect of therapies (within the last 2 months) on distress, the ANOVA revealed significant main effects for therapies (F=72.05, *P*<0.001) and gender (women>men; F=36.92, *P*<0.001), whereas there was no significant interaction.

Contrast analysis within ANOVA showed significant differences between no treatment group and patients receiving chemotherapy only, chemotherapy in combination with surgery and/or radiotherapy, or ‘other therapies’.

Table 5 shows mean distress scores in different therapy categories compared to the no treatment group stratified by gender. The analysis revealed the following results for men: patients with surgery showed the lowest scores similar to the no treatment group followed by hormone therapy and radiotherapy. The highest scores were reported by men with ‘other therapies’. In women, a similar pattern emerged: surgery and radiotherapy showed no difference compared to ‘no therapy’; highest distress score was reported for ‘other therapy’.

Finally, the effect of diagnostic groups on psychosocial distress was investigated in men and women. Significant main effects were found for diagnosis (F=11.28, *P*<0.001) and gender (women>men; F=11.06, *P*=0.001), but there was no interaction effect.

Table 6 shows mean distress scores for males and females. In males, the highest scores were found in patients with cancer of the respiratory tract followed by haematological neoplasias; lowest scores were reported by ENT carcinoma patients; overall differences within men were significant, with *P*<0.001. Women with carcinomas of the respiratory tract showed the highest distress scores followed by gynaecological carcinomas; relatively low scores were found in women with breast cancer, ENT carcinomas, and skin cancer; overall differences were significant in women as well, with *P*<0.001. Although women reported slightly higher distress than men in every diagnostic group, none of these differences were significant after controlling for multiple comparisons.

## Discussion

This is the first study investigating psychooncological distress in a large sample of cancer patients with a cancer-specific expert rating scale. Most of the psychooncological literature is based on studies assessing the prevalence of mental disorders (categorical approach) (Derogatis, 1993; Härter *et al*, 2001), quality-of-life studies (Spilker, 1990; Cella *et al*, 1993), or studies using global or specific distress questionnaires (dimensional approach) (Zabora *et al*, 2001; Herschbach *et al*, 2004a; Bultz and Carlson, 2006). We believe that cancer-specific distress measures have the highest clinical relevance (for determining the need of support or planning of psychotherapeutic interventions). Furthermore, the use of expert rating scales has the advantage of examining aspects of experience that are not assessed by self-assessment questionnaires and that they can be used for patients who are unable to answer questionnaires.

In this study, we investigated the expert-rated global distress and analysed the relevance of three distress conditions: setting variables, gender, and diagnosis.

Overall, female patients were more distressed than male patients, which supports findings from previous research (Zabora *et al*, 2001; Herschbach *et al*, 2004a; Bultz and Carlson, 2006). Looking at the analysis in more detail, women reported higher levels of distress in most of the psychological dimension whereas men had higher distress scores in the physical dimension.

To analyse the relevance of the treatment setting for psychosocial distress, the following variables were examined: type of clinic, contact initiative, and type of treatment. Looking at the type of clinic, patients receiving palliative treatment (outpatients care, palliative care ward, and hospice) had the highest global distress scores, which may be related to the severity of disease (somatic comorbidity). Furthermore, there was a significant interaction between type of clinic and gender. Thus, female patients were more distressed than male patients in university and rehabilitation clinics. A possible reason for this finding may be complex confounding effects of diagnosis and stage of disease in these settings.

Additionally, the way patients were selected to participate in this study (initiated by the patient, by medical staff, or by routine assessment) had a significant effect on psychological distress. As expected, patients who were recruited through routine contact had the lowest stress scores. Interestingly, only when patient contact was initiated by the medical staff, there was no gender difference. Otherwise, females showed higher levels of distress than males. Obviously, gender is not a variable that has a major impact on the distress assessment of the staff.

In terms of medical treatment, we compared single and combined medical treatment procedures with ‘no therapy’. Thus, receiving surgery or radiotherapy (within the last 2 months) is not more distressing than having no treatment. This is also true for men with regard to hormone therapy and for women with surgery plus radiotherapy. Receiving no treatment may be much more distressing. Highest distress was found in patients with ‘other therapies’. Assuming that ‘other therapies’ are complementary or experimental therapies, it is likely that those patients do not have curative treatment options any more. Besides these therapies, chemotherapy is the most distressing procedure. Combinations of therapy such as radio-chemotherapy are not necessarily more distressing than single intervention alone.

Looking at the role of diagnostic subgroups and gender, we found significant main effects for diagnosis and gender but no significant interaction. Patients with gynaecological carcinomas and cancer of the respiratory tract reported the highest levels of distress. In terms of gender, female patients were generally more distressed than male patients but did not differ significantly within the diagnostic subgroups.

Altogether, the study showed that type of treatment has an important impact on psychological distress of cancer patients. However, this does not imply that treatment in general is more distressing than no treatment. Certain types of treatment (surgery, hormone therapy, and radiotherapy) may be related to the hope of survival and may therefore be associated with lower levels of distress. This finding supports previous research on quality of life indicating that objective illness and treatment variables are only weakly correlated with the subjective illness experience (Herschbach, 2002). The second major result refers to the role of gender. The literature suggests that female cancer patients are generally more distressed than male patients (Zabora *et al*, 2001; Herschbach *et al*, 2004a). This suggestion is partly supported by the current study, as females reported higher overall distress; however, looking at the different dimensions of distress, we found that females are more distressed than males only in terms of psychological distress and not in terms of somatic distress. Relating gender to diagnostic subgroups, we did not find any significant gender differences.

There are some methodological limitations in our study that should be mentioned. The sample included 6365 cancer patients from a multicentre study including 105 institutions, and was associated with the following problems.

First, patients were selected in three different ways: they asked for psychosocial support, they were contacted by doctors or nurses, or they were recruited through routine contact. Thus, patients were recruited consecutively only for routine contact and there may be a selection bias for the other two types of recruitment.

Second, the main aim of our study was the influence of setting variables and gender on psychosocial distress; we are aware that the second aim, the importance of diagnosis, would not be representative in its distribution across the sample.

Finally, it would be interesting to analyse the influence of staff using the rating scale (discipline, job experience, interviewer training, etc.); however, owing to the large and heterogenic sample, this information is not available and should be the subject of future research.

We believe that there are good reasons to use expert rating scales; some of the new and unexpected findings of this study may be based on the use of this scale.

## Figures and Tables

**Table 1:** Demographic characteristics of patients

	**Male**		**Female**		**Total**	
	***N***	**%**	***N***	**%**	***N***	**%**
	**2177**	**34**	**4162**	**66**	**6356**	**100**
*Age, mean (s.d.)*						
Years	60.1 (14.3)		59.2 (13.8)		59.5 (14.0)	
Range	18–98		16–99		16–99	
						
*Age groups*						
<40 years	229	11	396	10	627	10
41–50 years	249	12	746	18	997	16
51–60 years	429	20	951	23	1385	22
61–70 years	714	33	1133	28	1849	30
>70 years	519	24	890	22	1413	22
						
*With partner* a						
Yes	1128	79	2284	69	3412	71
						
*Employment* a						
Employed	80	6	330	10	410	9
Sick leave	456	32	942	28	1398	30
Retired	804	56	1355	41	2159	45
Homemaker	5	0	500	15	505	11
Unemployed	53	4	93	3	146	3
Other	35	2	89	3	124	2

a Not available for HOPE study patients.

**Table 2:** Medical characteristics of patients

	**Male**		**Female**		**Total**	
	***N***	**%**	***N***	**%**	***N***	**%**
*Tumour site*						
Breast	8	0.4	2174	52	2182	34
ENT carcinomas	189	9	59	1	248	4
Haematological neoplasias	420	19	324	8	745	12
Upper gastrointestinal tract	289	13	250	6	540	9
Lower gastrointestinal tract	283	13	255	6	541	9
Gynaecological carcinomas	0	0	515	12	515	8
Respiratory tract	279	13	171	4	457	7
Male genito-urinary tumours	303	14	0	0	303	5
Urinary tract	91	4	69	2	160	3
Sarcomas	87	4	86	2	174	3
Skin cancer	41	2	46	1	87	1
CUP; rare diagnoses; others	187	9	213	5	404	6
						
						
Metastases	1087	50	1629	39	2716	43
*State of disease* a						
First occurrence	769	54	2198	66	2967	63
Recurrence	317	22	674	20	991	21
Second tumour	46	3	164	5	210	4
Remission	211	15	160	5	371	8
Unknown	90	6	111	3	201	4
						
*Duration of illness, mean (s.d.)* a						
Months	22.2 (42.5)		22.0 (49.6)		22.1 (47.7)	
Range	1 month–38 years		1 month–41 years		1 month–41 years	
						
*Categories*						
<1 month	245	20	1224	40	1469	34
2–12 months	570	46	963	31	1533	36
1–5 years	300	24	538	18	838	19
>5 years	132	11	337	11	469	11
						
*Treatment during the last 2 months* a						
Surgery	190	14	1234	38	1424	30
Chemotherapy	443	32	631	19	1074	23
Radiotherapy	62	4	83	3	145	3
Surgery+chemotherapy	37	3	139	4	176	4
Surgery+radiotherapy	52	4	155	5	207	4
Chemotherapy+radiotherapy	129	9	217	7	346	7
Surgery+chemotherapy+radiotherapy	27	2	49	1	76	2
Hormone therapy	6	0.4	119	4	125	3
Other therapies	93	7	143	4	236	5
No therapy	367	26	499	15	866	19
						
*Contact initiative* a						
Patient	276	19	883	27	1159	24
Medical staff	364	25	892	27	1256	27
Routine	793	55	1534	46	2327	49
						
*Treatment setting*						
University hospital	840	39	1434	34	2274	36
General hospital	466	21	1595	38	2061	32
Rehabilitation clinic	118	5	233	6	351	6
Outpatient clinic	9	0.4	48	1	57	1
Outpatient palliative care	136	6	161	4	300	5
Palliative care ward	545	25	595	14	1150	18
Hospice	63	3	96	2	163	3

a Not available for HOPE-study patients.

**Table 3:** Total distress scores for type of clinic

	**Male**		**Female**		**Total**		
	**Mean**	**s.d.**	**Mean**	**s.d.**	**Mean**	**s.d.**	P*
University hospital	15.12	9.89	19.81	10.01	18.08	10.22	<0.001
General hospital	13.63	7.20	12.59	7.20	12.83	7.21	NS
Rehabilitation clinic	10.46	7.56	16.59	9.19	14.53	9.14	<0.001
Outpatient clinic	13.44	9.62	17.73	9.67	17.05	9.71	NS
Outpatient palliative care	19.78	10.30	20.21	8.59	20.15	9.46	NS
Palliative care ward	21.31	8.54	22.27	8.66	21.84	8.62	NS
Hospice	20.94	9.66	21.99	8.33	21.35	8.90	NS

^*^Significance for the differences between male and female patients within each type of clinic; *P*-values corrected for multiple testing.

**Table 4:** Total distress scores for contact initiative

	**Male**		**Female**		**Total**		
	**Mean**	**s.d.**	**Mean**	**s.d.**	**Mean**	**s.d.**	P*
Patient	16.86	8.63	19.46	9.74	18.84	9.55	<0.001
Medical staff	19.30	9.64	18.98	9.61	19.07	9.62	NS
Routine	11.02	7.33	12.44	7.49	11.95	7.46	<0.001

^*^Significance for differences between male and female patients within each category of contact initiative; *P*-values corrected for multiple testing.

**Table 5:** Total distress scores for male and female patients in subgroups of therapy compared to no treatment

	**Male**			**Female**			**Total**		
	**Mean**	**s.d.**	*P*1*	**Mean**	**s.d.**	*P*2*	**Mean**	**s.d.**	*P*3*
**No treatment**	**10.09**	**7.94**		**13.82**	**8.93**		**12.24**	**8.72**	**<0.001**
Surgery	9.42	6.95	NS	13.33	8.50	NS	12.81	8.42	<0.001
Chemotherapy	18.44	8.72	<0.001	20.86	9.26	<0.001	19.86	9.11	<0.001
Radiotherapy	12.10	8.17	NS	16.45	9.46	NS	14.59	9.16	NS
Hormone therapy	11.17	5.88	NS	17.99	9.72	<0.001	17.66	9.66	NS
Other therapy	19.38	9.88	<0.001	22.04	9.78	<0.001	20.99	9.89	NS
Surgery+chemotherapy	18.46	9.66	<0.001	19.10	9.37	<0.001	18.97	9.41	NS
Surgery+radiotherapy	13.37	5.48	<0.001	14.21	7.28	NS	14.00	6.87	NS
Chemotherapy+radiotherapy	15.12	7.62	<0.001	16.51	8.12	<0.001	15.99	7.96	NS
Surgery+chemotherapy+radiotherapy	14.93	5.66	<0.001	19.76	8.47	<0.001	18.04	7.90	NS

^*^P1: difference between each therapy group and the non-treatment group within males; *P*2: difference between each therapy group and the non-treatment group within females; *P*3: differences between male and female patients within each therapy group; ^*^P-values corrected for multiple testing.

**Table 6:** Total distress scores for diagnostic subgroups

	**Male**		**Female**		**Total**		
	**Mean**	**s.d.**	**Mean**	**s.d.**	**Mean**	**s.d.**	P*
Breast	16.00	10.39	15.70	9.22	15.72	9.24	NS
Gynaecological carcinomas			20.10	9.30	20.13	9.31	
Respiratory tract	19.15	9.19	20.57	9.01	19.76	9.13	NS
Male genito-urinary tumours	15.16	9.70			15.13	9.68	
Lower gastrointestinal tract	14.55	9.45	16.20	9.24	15.39	9.39	NS
ENT carcinomas	12.24	9.29	15.88	11.01	13.11	9.83	NS
Haematological neoplasias	18.33	8.63	19.25	9.15	18.74	8.86	NS
Skin cancer	15.66	9.24	16.30	9.37	16.00	9.26	NS
Sarcomas	16.54	10.32	19.56	9.87	18.03	10.15	NS
Urinary tract	16.37	10.16	18.30	9.86	17.21	10.05	NS
Upper gastrointestinal tract	17.92	9.45	19.43	9.05	18.61	9.29	NS
Others	16.43	9.48	18.48	10.33	17.58	10.03	NS

^*^Differences between male and female patients within diagnostic subgroups; *P*-values corrected for multiple testing.
